# Physiologic Factors Influencing the Arterial-To-End-Tidal CO_2_ Difference and the Alveolar Dead Space Fraction in Spontaneously Breathing Anesthetised Horses

**DOI:** 10.3389/fvets.2018.00058

**Published:** 2018-03-28

**Authors:** Martina Mosing, Stephan H. Böhm, Anthea Rasis, Giselle Hoosgood, Ulrike Auer, Gerardo Tusman, Regula Bettschart-Wolfensberger, Johannes P. Schramel

**Affiliations:** ^1^College of Veterinary Medicine, Murdoch University, Perth, WA, Australia; ^2^Department of Anesthesiology and Intensive Care Medicine, Rostock University Medical Center, Rostock, Germany; ^3^Anaesthesiology and Perioperative Intensive Care Medicine, Veterinary University Vienna, Vienna, Austria; ^4^Department of Anesthesiology, Hospital Privado de Comunidad, Mar del Plata, Argentina; ^5^Division of Anaesthesiology, Vetsuisse Faculty, Zurich, Switzerland

**Keywords:** equine, spontaneous ventilation, volumetric capnography, airway dead space, pulmonary perfusion

## Abstract

The arterial to end-tidal CO_2_ difference (P_(a-ET)_CO_2_) and alveolar dead space fraction (VDalv_frac_ = P_(a-ET)_CO_2_/PaCO_2_), are used to estimate Enghoff’s “pulmonary dead space” (V/Q_Eng_), a factor which is also influenced by venous admixture and other pulmonary perfusion abnormalities and thus is not just a measure of dead space as the name suggests. The aim of this experimental study was to evaluate which factors influence these CO_2_ indices in anesthetized spontaneously breathing horses. Six healthy adult horses were anesthetized in dorsal recumbency breathing spontaneously for 3 h. Data to calculate the CO_2_ indices (response variables) and dead space variables were measured every 30 min. Bohr’s physiological and alveolar dead space variables, cardiac output (CO), mean pulmonary pressure (MPP), venous admixture $\left(\dot{Q}s\text{​}/\text{​}\dot{Q}t\right)$, airway dead space, tidal volume, oxygen consumption, and slope III of the volumetric capnogram were evaluated (explanatory variables). Univariate Pearson correlation was first explored for both CO_2_ indices before V/Q_Eng_ and the explanatory variables with rho were reported. Multiple linear regression analysis was performed on P_(a-ET)_CO_2_ and VDalv_frac_ assessing which explanatory variables best explained the variance in each response. The simplest, best-fit model was selected based on the maximum adjusted *R*^2^ and smallest Mallow’s p (C_p_). The *R*^2^ of the selected model, representing how much of the variance in the response could be explained by the selected variables, was reported. The highest correlation was found with the alveolar part of V/Q_Eng_ to alveolar tidal volume ratio for both, P_(a-ET)_CO_2_ (*r* = 0.899) and VDalv_frac_ (*r* = 0.938). Venous admixture and CO best explained P_(a-ET)_CO_2_ (*R*^2^ = 0.752; C_p_ = 4.372) and VDalv_frac_ (*R*^2^ = 0.711; C_p_ = 9.915). Adding MPP (P_(a-ET)_CO_2_) and airway dead space (VDalv_frac_) to the models improved them only marginally. No “real” dead space variables from Bohr’s equation contributed to the explanation of the variance of the two CO_2_ indices. P_(a-ET)_CO_2_ and VDalv_frac_ were closely associated with the alveolar part of V/Q_Eng_ and as such, were also influenced by variables representing a dysfunctional pulmonary perfusion. Neither P_(a-ET)_CO_2_ nor VDalv_frac_ should be considered pulmonary dead space, but used as global indices of V/Q mismatching under the described conditions.

## Introduction

Ventilation/perfusion (V/Q) mismatch develops rapidly in anesthetized horses (1). Venous admixture and pulmonary dead space mark the two “extremes” of the spectrum of this mismatch; the former representing perfused but not ventilated alveoli (V/Q = 0) while the latter representing alveoli which are only ventilated but not perfused (V/Q = ∞).

Several oxygen indices including arterial-to-alveolar partial pressure of oxygen (PO_2_) difference and P_a_O_2_/inspiratory fraction of oxygen (FiO_2_) ratio or f-shunt have been tested to estimate venous admixture in people and horses (2) and are frequently applied in clinical routine. The index used to estimate physiologic dead space is the difference between the arterial and end-tidal partial pressure of CO_2_ [P_(a-ET)_CO_2_], which was evaluated in spontaneously breathing and ventilated people several decades ago (3). The P_(a-ET)_CO_2_ was divided by P_a_CO_2_ and denoted as an estimate of alveolar dead space, and called alveolar dead space fraction [VDalv_frac_ = P_(a-ET)_CO_2_/P_a_CO_2_] (4). The concept of using P_a_CO_2_ and P_ET_CO_2_ to estimate “dead space” was adapted in equine anesthesia over 40 years ago (5, 6) and has been used extensively ever since to estimate “dead space” in many animal species (7).

“True” dead space measurements, however, include the measurement of mixed-expired partial pressure of CO_2_ (P_Ē_CO_2_) and the mean alveolar CO_2_ (P_A_CO_2_) (8). Bohr used these two CO_2_ measurements to calculate the famous physiologic dead space ratio [VD_Bohr_ = (P_A_CO_2_ − P_Ē_CO_2_)/P_A_CO_2_]. As P_A_CO_2_ was difficult to obtain, Enghoff suggested more than 50 years later that the partial pressure of CO_2_ in the arterial blood would be representative of the P_A_CO_2_. Therefore, he modified Bohr’s formula substituting P_A_CO_2_ by P_a_CO_2_ to derive the following formula: VD_Eng_ = (P_a_CO_2_ − P_Ē_CO_2_)/P_a_CO_2_ (9). What Enghoff failed to account for is the fraction of shunting blood volume that cannot unload CO_2_ during lung passage and thereby adding CO_2_-rich blood to the arterial side (10). This fact is the reason why P_a_CO_2_ almost invariably exceeds P_A_CO_2_. The contribution of this blood flow equals venous admixture and has been shown to cause VD_Eng_ to overestimate the “true” physiologic dead space (11–13). On a positive note, it should be used as a global index of V/Q mismatching since VD_Eng_ includes pulmonary dead space and venous admixture alike (10, 12). It is important to note that all studies concluding that P_(a-ET)_CO_2_ and VDalv_frac_ is valid representative of pulmonary dead space used VD_Eng_ as their reference values for dead space (3–5). As VD_Eng_ does not represent the “true” physiologic dead space, it is not surprising that more recent studies show the unreliability of P_(a-ET)_CO_2_ and VDalv_frac_ to predict dead space or changes thereof (14–16). Following aforementioned assumptions the main factors causing an inaccuracy in dead space calculation using P_(a-ET)_CO_2_ and VDalv_frac_ are venous admixture and atelectasis formation (13, 17, 18). Furthermore, other physiologic factors like tidal volume, oxygen consumption, respiratory rate, and homogeneity of lung emptying have been shown to change the difference between the arterial and the end-tidal CO_2_ values (14, 15, 19). To account for the fact that Enghoff’s “dead space” is influenced by V/Q = ∞, V/Q = 0 and the global V/Q mismatch, for clarity, the term will be referred to as Enghoff’s approach (V/Q_Eng_) in this manuscript.

**Figure 1:**
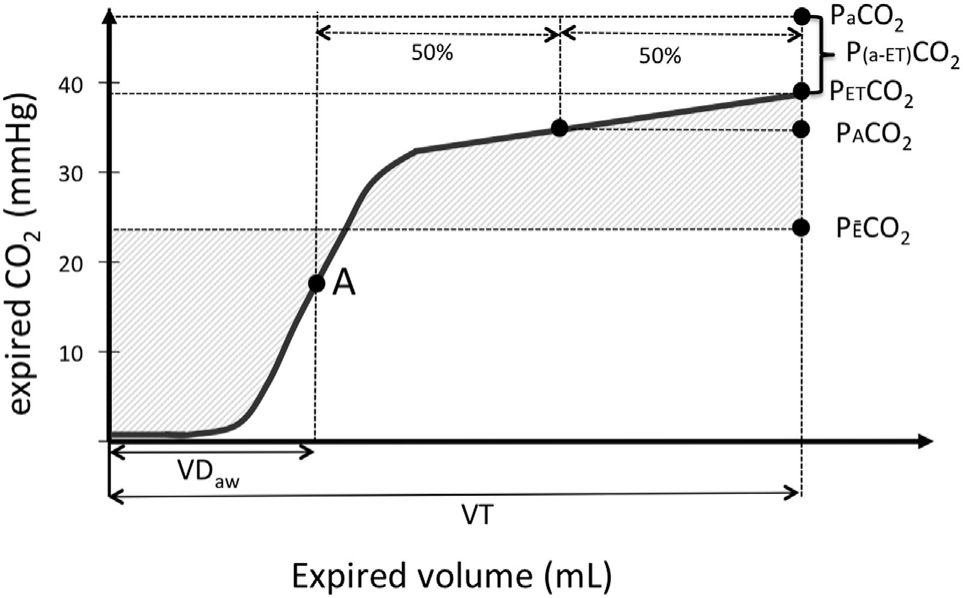
Graphical illustration of a volumetric capnography (VCap) curve where the partial pressure of expired CO_2_ (Exp. CO_2_) is plotted over the volume of one tidal breath (VT). “A” is the inflection point of VCap that separates per definition the airways from alveolar compartment and is used to calculate airway dead space (VDaw). PACO_2_ is the CO_2_ value at the midpoint between the inflection point “A” and the end-tidal CO_2_ (P_ET_CO_2_). The mixed-expired CO_2_ (P_E¯_CO_2_) represents the mean CO_2_ in the expired gas. The difference between P_ET_CO_2_ and arterial oxygen partial pressure (PaCO_2_) is plotted as well.

Based on the above considerations, there is a clinical need for a reliable and yet easy to obtain alternative index of dead space overcoming the time consuming difficult measurement of CO_2_ variables needed for Bohr’s equation. Until two decades ago, mixed-expired CO_2_, which is employed in Bohr’s equation and Enghoff’s approach, could only be measured by using a mixing chamber or Douglas bag (20). More recently, volumetric capnography (VCap) provides an easier and clinically more convenient way to measure P_Ē_CO2. To obtain the VCap curve the partial pressure of CO_2_ is plotted over the expired tidal volume—therefore the term VCap (13). P_Ē_CO_2_ is the average of the partial pressure of CO_2_ during exhalation. It is calculated from the area under the curve (the integral of the VCap curve during exhalation) divided by tidal volume (10). Beside P_Ē_CO_2_ also P_A_CO_2_ and airway dead space can be derived from the VCap curve (Figure 1) (21). For this evaluation, the exhaled gas flow and partial pressure of CO_2_ needs to be measured simultaneously. Due to the time delay of the CO_2_ sensor in side-stream capnography, a mainstream CO_2_ sensor combined with a pneumotachograph close to the endotracheal tube (ETT) is the most commonly used set-up to measure VCap. The VCap monitors developed for humans cannot cope with the high inspiratory and expiratory flow rates in horses. In order to allow the use of these pneumotachographs during equine anesthesia a custom-made flow-partitioning device can be inserted between ETT and the Y-piece of the anesthetic breathing circle (22). This device splits the flow into four equal parts, making it possible to calculate the correct volume by multiplying the measured values by four.

The aim of this study was to discern factors that best explain the variability in P_(a-ET)_CO_2_ and VDalv_frac_ in anesthetized spontaneously breathing horses in dorsal recumbency. We hypothesized that: (1) P_(a-ET)_CO_2_ and VDalv_frac_ is strongly associated with V/Q_Eng_ and derived variables, but much less with “true” dead space variables derived from Bohr’s original formula, (2) P_(a-ET)_CO_2_ and VDalv_frac_ is highly influenced by venous admixture and other factors influencing lung perfusion.

## Materials and Methods

This experimental study had ethical approval by the local committee for animal experimentation of the Swiss government (TV-4985). It was part of an original study performed to evaluate the effects of CPAP in anesthetized horses (23). Only those horses in which no CPAP was applied were included in this current study.

### Animals

Six horses were included in this experimental study out of the control group of a former study (23). Horses were acquired from local breeders and housed in boxes at the hospital for at least a week before commencing this study. All horses were considered healthy based on thorough clinical examination, routine blood work, and arterial blood gas analysis before anesthesia. They were fasted for 12 h, but had free access to water until 2 h prior to induction.

Before premedication, a 8F 110-cm angiography balloon catheter (Arrow Swiss, Edwards Lifesciences, Switzerland) was placed *via* the jugular vein in the pulmonary artery using pressure guidance to determine correct positioning.

### Anesthesia

Premedication consisted of medetomidine (0.007 mg kg^−1^) (Dorbene, Graeub AG, Switzerland) IV and phenylbutazone (4 mg kg^−1^) IV (Butadion, Streuli Pharma AG, Switzerland). Anesthesia was induced with diazepam (0.02 mg kg^−1^) (Valium, Roche, Switzerland) and ketamine (2 mg kg^−1^) (Ketanarkon 100, Streuli Pharma AG, Switzerland) IV. After induction, a 26-mm internal diameter cuffed silicone ETT was inserted into the trachea and connected to a circle breathing system (Tafonius, Vetronic Services Ltd., UK) after placing the horse on a mattress in dorsal recumbency. Anesthesia was maintained using isoflurane (Attane Isoflurane, Provet, Switzerland) in an oxygen/air mixture (inspiratory O_2_ fraction: FiO_2_ = 0.5). Medetomidine was infused as a continuous rate infusion (0.0035 mg kg^−1^ h^−1^). Ketamine (50–100 mg bolus) was administered IV in case of spontaneous movement.

Two multi-parameter monitors (Datex-Ohmeda S/3 Anesthesia Monitor, Datex-Ohmeda and Solomon monitoring system, Vetronics, UK) were used to measure standard cardiopulmonary parameters throughout anesthesia as well as mean pulmonary artery pressure. The pressure transducer was zeroed to atmospheric pressure and leveled at the height of the shoulder joint. New pressure transducers were used for each horse.

Ringer’s lactate (Ringer-Lactat Fresenius, Fresenius Kabi, Switzerland) was infused at a rate of 10 mL kg^−1^ h^−1^ and hydroxyethyl starch (HAES-steril 10% ad us. vet., Fresenius Kabi, Switzerland) was started 2 h after induction (1 mL kg^−1^ h^−1^) to account for the expected fluid losses due to medetomidine. Dobutamine was infused intravenously to maintain mean arterial pressure between 75 and 85 mmHg.

If arterial PCO_2_ (P_a_CO_2_) exceeded 100 mmHg (13.3 kPa) at any measurement point horses were excluded from further data acquisition and mechanical ventilation was started.

In the original study all horses were anesthetized for 6 h but only data from the first 3 h were used in this study. At the end of the study period, horses were allowed to recover unassisted.

### Monitoring and Data Collection

All cardiovascular data including mean pulmonary pressure (MPP) were recorded every 30 min during the first 3 h of anesthesia (T30, T60, T90, T120, T150, T180). Cardiac output (CO) was measured in triplicate at T60, T120, and T180 using the lithium dilution technique (LiDCO; LiDCO Group, UK) (24).

A flow-partitioning device was placed between the ETT and the Y-piece of the circle system dividing the total flow into four parts by two flow splitting adapters connected to each other by means of four silicon tubes equipped with four identical human adult NICO capnograph connectors (Respironics, Wallingford, CT, USA) (22). These connectors include a fixed-orifice resistance for flow measurement and a connection site for the mainstream sensor of the capnography unit of the NICO. Only one of the four connectors was connected to the spirometer and mainstream capnography unit of the NICO.

At time points of cardiovascular evaluation, VCap and spirometry data were recorded for 3 min using the dedicated software Datacoll (Respironics, Wallingford, CT, USA). Furthermore, simultaneous arterial and mixed venous blood samples were taken under anaerobic conditions at each of these time points and PO_2_, carbon dioxide tension (PCO_2_) and arterial hemoglobin concentration ([Hb]) analyzed immediately (Rapidpoint, Siemens, Switzerland).

All devices were calibrated following manufacturer’s guidelines before each anesthesia; the capnograph of the NICO device was calibrated with room air before each experiment (infrared sensor with a response time <60 ms and accuracy of ±2 mmHg) and the accuracy of the pneumotachograph was verified with a 100-mL calibration syringe before and after all measurements (allowed accuracy of ±3% following manufacturers guidelines for the calibration set-up). The accuracy of the volume measurement of the flow-partioning device used in our set-up in connection with the NICO device was verified in an *in vitro* experiment published elsewhere (25). The difference between the reading of the flow-partitioning device in combination with the NICO device and the 10 L calibration syringe was 0.73 ± 4.3% (mean ± SD).

### Data Analysis

After importing CO_2_ values into an Excel spread sheet, P_(a-ET)_CO_2_ and VDalv_frac_ were calculated. VCap data were imported into a custom-made macro routine in Excel (Excel; Microsoft Corporation, WA, USA) and the recorded breaths analyzed. The mean value of all recorded breaths per time point and horse was used for statistical analysis. Bohr’s dead space ratio (VD_Bohr_) and V/Q_Eng_ were calculated following curve approximation by the solver function according to Tusman et al. (26). The ratio of alveolar dead space (difference between physiologic VDs and VD_aw_) to alveolar tidal volume was calculated based on Bohr’s (VD_alv_Bohr/VT_alv_) and Enghoff’s (V/Q_alv_Eng/VT_alv_) respective equations. Airway dead space was defined as the volume at the inflection point of the VCap curve and was normalized by the tidal volume (VD_aw_/VT). The slope of phase III of the volumetric capnogram (SIII) was determined. Venous admixture $\left(\dot{Q}s\text{​}/\text{​}\dot{Q}t\right)$ and oxygen consumption (VO_2_) were calculated using standard equations (27).

### Statistics

Continuous data were summarized and reported as mean ± SD and range. All continuous variables were deemed to follow a normal distribution based on Q–Q plots and failure to reject the null hypothesis of normality was tested using Shapiro–Wilk’s statistic at *P* ≤ 0.05.

For the purpose of explaining the variance of the responses P_(a-ET)_CO_2_ and VDalv_frac_, the explanatory variables were grouped according to what physiologic state they best represented; VD_Bohr_, VD_alv_Bohr/VT_alv_, and VD_aw_/VT were considered “true” dead space variables; CO and MPP were considered variables related to pulmonary blood flow; $\dot{Q}s\text{​}/\text{​}\dot{Q}t$ was considered a functional variable mirroring collapsed lung areas with low V/Q causing CO_2_ admixture to the arterial side; SIII was treated as a factor characterizing homogenity of lung emptying; and VT and VO_2_ were considered possible explanatory variables based on evidence in the literature.

Initial explorations of the association between Enghoff’s variables (V/Q_Eng_, V/Q_alv_Eng/VT_alv_) and P_(a-ET)_CO_2_ and VDalv_frac_ were performed using Pearson’s correlation with rho reported for each variable. All data points from each horse were used. Multiple linear regression analysis was then performed on P_(a-ET)_CO_2_ and VDalv_frac_, assessing all combinations of explanatory variables to determine which explanatory variables best explained the variance of each response. The simplest, best-fit model was selected based on the maximum adjusted *R*^2^ and the smallest Mallow’s p (C_p_). The *R*^2^ of the selected model, representing how much of the variance in the response could be explained by the variance of the selected variables, was reported. Enghoff’s approach and the associated variables (representing high and low V/Q ratios) were not included in the multi-variate analysis as the aim of this analysis was to show if the two indices represent V/Q = ∞ and areas of high V/Q ratio, or V/Q = 0 and areas of low V/Q ratio.

## Results

Six healthy adult horses with a mean (SD) age of 10 ± 6 years and body weight of 539 ± 35 kg (one Thoroughbred, five Freiberger) completed the 3 h study period and complete data sets of all horses could be analyzed.

Respiratory variables, VCap, and specific cardiovascular data of all horses are given in Table 1.

**Table 1:** Mean ± SD of the arterial to end-tidal CO_2_ difference, alveolar dead space fraction, dead space and cardiorespiratory parameters in six spontaneously breathing horses under general isoflurane anesthesia in dorsal recumbency at different time points (T30–180).

	T30	T60	T90	T120	T150	T180
P_(a-ET)_CO_2_	13.14 ± 8.42	11.15 ± 6.90	12.07 ± 4.34	13.39 ± 4.95	21.93 ± 8.89	21.40 ± 8.97
VDalv_frac_	0.20 ± 0.12	0.17 ± 0.09	0.19 ± 0.05	0.20 ± 0.07	0.27 ± 0.08	0.26 ± 0.07
P_E¯_CO_2_	48.54 ± 3.96	49.42 ± 3.59	51.80 ± 4.41	54.02 ± 4.37	56.57 ± 4.14	58.91 ± 8.13
PaCO_2_	61.68 ± 8.90	58.66 ± 6.55	63.87 ± 7.29	67.33 ± 5.79	77.43 ± 10.85	80.32 ± 14.49
ETCO_2_	48.54 ± 3.93	49.42 ± 3.59	51.80 ± 4.41	54.02 ± 4.37	56.57 ± 4.14	58.91 ± 8.13
V/Q_Eng_	0.55 ± 0.06	0.49 ± 0.01	0.52 ± 0.04	0.51 ± 0.03	0.56 ± 0.06	0.57 ± 0.06
V/Q_alv_Eng/VT_alv_	0.26 ± 0.11	0.23 ± 0.07	0.26 ± 0.04	0.27 ± 0.06	0.33 ± 0.09	0.33 ± 0.06
VD_Bohr_	0.39 ± 0.08	0.35 ± 0.04	0.37 ± 0.04	0.35 ± 0.04	0.37 ± 0.05	0.38 ± 0.06
VD_alv_Bohr/VT_alv_	0.02 ± 0.02	0.03 ± 0.01	0.03 ± 0.01	0.04 ± 0.01	0.04 ± 0.01	0.03 ± 0.01
VD_aw_/VT	0.38 ± 0.10	0.33 ± 0.05	0.35 ± 0.04	0.33 ± 0.05	0.34 ± 0.05	0.36 ± 0.07
CO		28.16 ± 4.91		39.85 ± 6.64		46.10 ± 7.88
MPP	12.20 ± 4.87	14.50 ± 5.01	12.60 ± 7.70	14.33 ± 6.50	13.83 ± 5.34	12.00 ± 5.93
$\dot{Q}s/\dot{Q}t$	0.23 ± 0.11	0.28 ± 0.10	0.31 ± 0.11	0.32 ± 0.09	0.34 ± 0.11	0.34 ± 0.10
VO_2_		104 ± 16		115 ± 14		111 ± 18
VT	5,876 ± 2,135	6,342 ± 1,492	5,985 ± 631	6,470 ± 1,197	6,427 ± 1,245	6,085 ± 1,206
SIII	0.006 ± 0.006	0.003 ± 0.001	0.003 ± 0.001	0.002 ± 0.001	0.003 ± 0.001	0.004 ± 0.002

*P_(a-ET)_CO_2_ (mmHg), arterial to end-tidal CO_2_ partial pressure difference; VDalv_frac_, alveolar dead space fraction; P_Eˉ_CO_2_ (mmHg), mixed-expired partial pressure of CO_2_; PaCO_2_ (mmHg), arterial partial pressure of CO_2_; ETCO_2_ (mmHg), end-tidal partial pressure of CO_2_; V/Q_Eng_, Enghoff’s dead space ratio; V/Q_alv_Eng/VT_alv_, alveolar dead space as a ratio to alveolar tidal volume calculated from VD_Eng_; VD_Bohr_, Bohr’s dead space ratio; VD_alv_Bohr/VT_alv_, alveolar dead space as a ratio to alveolar tidal volume calculated from VD_Bohr_; VD_aw_/VT, airway dead space per tidal volume; CO (L/min), cardiac output; MPP (mmHg), mean pulmonary pressure; $\dot{Q}s\text{​}/\text{​}\dot{Q}t$, venous admixture; VO_2_ (mL/min), oxygen consumption; V_T_ (L), tidal volume; SIII (mmHg/mL), slope of phase III of the volumetric capnogram*.

The P_(a-ET)_ CO_2_ ranged from −1 to 39 mmHg with the highest values at T120 (one horse), T150 (two horses), and T180 (three horses).

**Figure 2:**
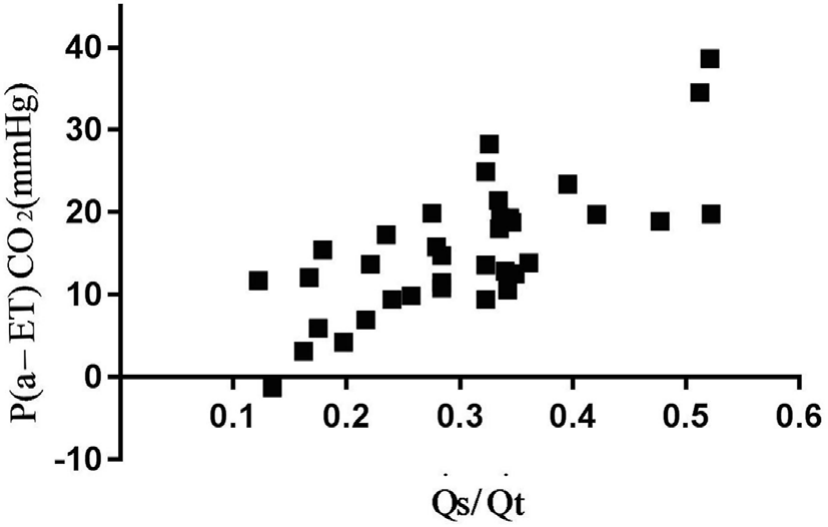
Association between arterial to end-tidal CO_2_ difference (P_(a-ET)_CO_2_) and venous admixture ($\dot{Q}s\text{​}/\text{​}\dot{Q}t$) in six spontaneously breathing horses under general isoflurane anesthesia in dorsal recumbency. The graph demonstrates the relation over a wide range of $\dot{Q}s\text{​}/\text{​}\dot{Q}t$ values.

There was a high correlation of V/Q_alv_Eng/VT_alv_ and a moderate correlation of V/Q_Eng_, $\dot{Q}s\text{​}/\text{​}\dot{Q}t$, and CO with P_(a-ET)_ CO_2_ (Figure 2; Table 2).

**Table 2:** Pearson’s correlation coefficient (rho) for the arterial to end-tidal CO_2_ difference and alveolar dead space fraction with dead space and cardiorespiratory parameters in six spontaneously breathing horses under general isoflurane anesthesia in dorsal recumbency.

	P_(a-ET)_CO_2_	VDalv_frac_
V/Q_Eng_	0.718	0.659
V/Q_alv_Eng/VT_alv_	0.899	0.938
VD_Bohr_	0.121	0.262
VD_alv_Bohr/VT_alv_	0.188	0.207
VD_aw_/VT	0.130	0.247
CO	0.773	0.288
Mean pulmonary pressure	0.161	0.159
$\dot{Q}s/\dot{Q}t$	0.694	0.684
VO_2_	0.045	0.084
VT	0.418	0.317
SIII	0.116	0.130

*Descriptions, see Table 1*.

The variance of P_(a-ET)_CO_2_ was best explained by $\dot{Q}s\text{​}/\text{​}\dot{Q}t$ and CO, accounting for 75% (*R*^2^ = 0.7516). Adding MPP to the model improved the explanation by additional 2% (*R*^2^ = 0.7860) (Table 3).

**Table 3:** The results of multi linear regression analysis for factors associated with the arterial to end-tidal CO_2_ difference (P_(a-ET)_CO_2_) and alveolar dead space fraction (VDalv_frac_) in six spontaneously breathing horses under general isoflurane anesthesia in dorsal recumbency.

Selected variable	P_(a-ET)_CO_2_			VDalv_frac_		
	*R*^2^	Adjusted *R*^2^	C_p_	*R*^2^	Adjusted *R*^2^	C_p_
$\dot{Q}s/\dot{Q}t$	0.626	0.603	10.62	0.632	0.609	13.92
CO	0.130	0.075	43.35	0.083	0.083	55.68
VD_aw_/VT	0.002	−0.060	51.75	0.049	0.049	58.28
MPP	0.192	0.142	39.24	0.138	0.138	51.47
$\dot{Q}s/\dot{Q}t$ + CO	0.752	0.718	4.37	0.711	0.673	9.91
$\dot{Q}s/\dot{Q}t$ + CO + MPP	0.786	0.740	4.10			
$\dot{Q}s/\dot{Q}t$ + CO + VD_aw_/VT				0.744	0.690	9.42

*Venous admixture $\left(\dot{Q}s\text{​}/\text{​}\dot{Q}t\right)$ and CO best explained the variance in the responses, based on the adjusted *R*^2^ and Mallow’s *p* (C_p_). The overall *R*^2^ of the selected model is reported, representing how much of the variance in the response is explained by the selected variables*.

*P_(a-ET)_CO_2_ (mmHg), arterial to end-tidal CO_2_ partial pressure difference; VDalv_frac_, alveolar dead space fraction; $\dot{Q}s/\dot{Q}t$, venous admixture; CO (L/min), cardiac output; VD_aw_/VT, airway dead space per tidal volume; MPP (mmHg), mean pulmonary pressure*.

VDalv_frac_ ranged from −0.02 to 0.36 with the highest values observed at the same time points as the highest P_(a-ET)_CO_2_, except in one horse. This horse had the highest P_(a-ET)_CO_2_ earlier than the others showing consistently lower COs than all other horses during the entire study period.

**Figure 3:**
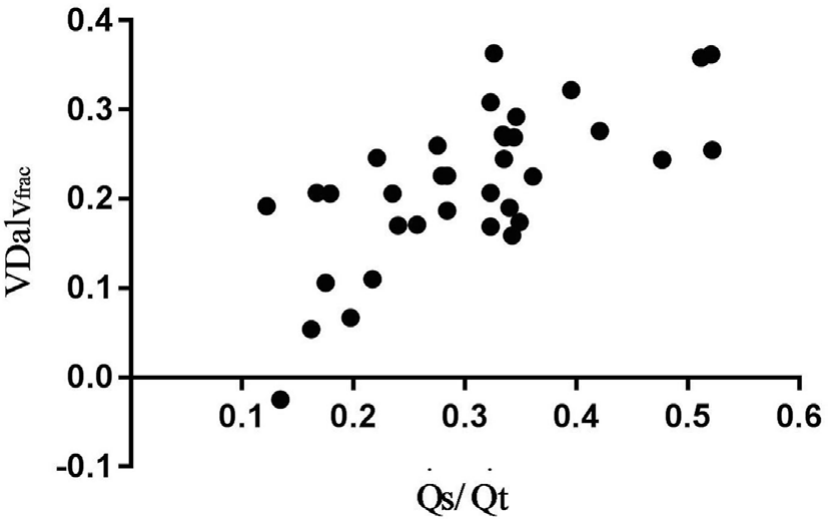
Association between alveolar dead space fraction (VDalv_frac_) and venous admixture ($\dot{Q}s\text{​}/\text{​}\dot{Q}t$) in six spontaneously breathing horses under general isoflurane anesthesia in dorsal recumbency. The graph demonstrates the relation over a wide range of $\dot{Q}s\text{​}/\text{​}\dot{Q}t$ values.

Correlation of VDalv_frac_ with V/Q_alv_Eng/VT_alv_ was high and moderate with V/Q_Eng_ and $\dot{Q}s\text{​}/\text{​}\dot{Q}t$ (Figure 3; Table 2).

The variance of VDalv_frac_ was best explained by $\dot{Q}s/\dot{Q}t$ and CO accounting for 71% of it (*R*^2^ = 0.7115). Adding VD_aw_/VT to the model improved the explanation by another 3% (*R*^2^ = 0.7444) (Table 3).

Neither Bohr’s dead space variables, nor SIII, VT, or VO_2_ showed any significant correlation or provided any further explanation of the variance of P_(a-ET)_CO_2_ and VDalv_frac_.

## Discussion

The results of this study support the first hypothesis in that both indices, P_(a-ET)_CO_2_ and VDalv_frac_ can be used to estimate Enghoff’s approach, but do not represent “true” dead spaces as derived from Bohr’s equation in spontaneously breathing horses. The results of this study also support the second hypothesis in that venous admixture and factors related to pulmonary perfusion, like CO and pulmonary pressure influence P_(a-ET)_CO_2_ and VDalv_frac_. Therefore, P_(a-ET)_CO_2_ and VDalv_frac_ did not represent V/Q = ∞ (physiologic or alveolar dead space) in these spontaneously breathing horses in dorsal recumbency, but a global mismatch in the pulmonary ventilation/perfusion ratio, a finding supported by studies in other species.

Looking at the difference between PaCO_2_ and ETCO_2_ from the theoretical side every increase of the difference can have two causes: (1) a decrease in ETCO_2_ relative to PaCO_2_ or (2) any increase in PaCO_2_ relative to ETCO_2_. In the following, we want to discuss the physiologic background for both causes related to the results found in this study.

(1) A drop in ETCO_2_ in relation to PaCO_2_ is indicative of an increase in ventilation of non-perfused (V/Q = ∞; alveolar dead space) or poorly perfused (areas of high V/Q ratio) alveoli, resulting in an increase in physiologic dead space. This fact directed Nunn and Hill (3) and Severinghaus et al. (4) toward the conclusion that physiologic or alveolar dead space could be estimated from these two parameters.

No drop in ETCO_2_ was seen in any of our horses at any time point. In fact ETCO_2_ increased continuously together with PaCO_2_ between the measurement points in all horses (Table 1). This indicates that physiologic dead space itself did not increase at all, or if so, only marginally. The VCap results confirmed this by showing only small changes in Bohr’s physiologic and alveolar dead space values throughout the study period. The relatively small amount of “real” physiologic and alveolar dead space and minimal changes could be expected in healthy horses with high CO under spontaneous ventilation. We consider this the most likely reason why we did not find any interaction between the two CO_2_ indices and our measured dead space variables. However, in states of low CO, overdistension of alveolar tissue during positive pressure ventilation, excessive PEEP or pulmonary thromboembolism the amount of well-ventilated but poorly or non-perfused alveoli (high V/Q and high Bohr’s alveolar dead space) will increase (10, 19, 27–29). These changes also affect V/Q_Eng_ as a factor for the global V/Q mismatch including dead space. This has been shown in a horse suffering from venous air embolism where V/Q_Eng_ nearly doubled during the unintended air infusion (29). As the two CO_2_ indices highly correlated with V/Q_Eng_, we can assume that in situations of high V/Q and high Bohr’s alveolar dead space P_(a-ET)_CO_2_ and VDalv_frac_ will also increase. Future studies will need to verify this assumption.

The only dead space variable with a relevant influence on VDalv_frac_ in our study was airway dead space. This parameter, which neither influences Enghoff’s nor Bohr’s equation, is derived from the VCap curve using mathematical modeling of the expired CO_2_ over the measured volume (26). Interestingly this was not the first time airway dead space was identified as a factor influencing P_(a-ET)_CO_2_ and VDalv_frac_; in a model simulating human physiology airway dead space was detected as one of two main factors, the second one being venous admixture, causing inaccuracies in the calculation of alveolar dead space when using these CO_2_ indices (15). As our horses were breathing spontaneously, only small changes in VD_aw_/VT were expected. However, the impact of VD_aw_/VT might become more relevant when using positive pressure ventilation, especially in combination with positive end-expiratory pressure, which has been shown by Lumb and others to increase airway dead space (27, 28). Thus, the increase in VD_aw_/VT can possibly increase VDalv_frac_ under controlled mechanical ventilation.

(2) The second change, which can influence the difference between PaCO_2_ and ETCO_2_, namely an increase in PaCO_2_ in relation to ETCO_2_, occurs when CO_2_ cannot be eliminated from the blood into the alveoli. This is the case when the blood flows through atelectatic lung tissue or areas with low V/Q ratios. This fact makes P_(a-ET)_CO_2_ and VDalv_frac_ highly variable in situations of high venous admixture (15, 30, 31). Our results confirmed the direct influence of venous admixture on P_(a-ET)_CO_2_ and VDalv_frac_ in horses with a broad range of venous admixture volumes. As horses develop extensive venous admixture during anesthesia (1), this species is well suited to show the direct influence of this pathological lung condition on the two CO_2_ indices in question. Our horses had between 12 and 52% of their CO perfusing poorly or non-ventilated lung units (V/Q = 0; venous admixture). This spectrum represents the expected amount of venous admixture in anesthetized horses in dorsal recumbency (32). This large difference in venous admixture was reflected by a wide range of P_(a-ET)_CO_2_ and VDalv_frac_ values. Strang et al. suggested that P_(a-ET)_CO_2_ can be used as an index of oxygenation as it correlated well with the amount of atelectasis in pigs seen on computed tomography being even more predictive of the amount of atelectasis than the most accepted oxygen index PaO_2_/FiO_2_ ratio (18). The strong relationship between P_(a-ET)_CO_2_ and an impaired oxygenation has also been shown by means of pulse oximetry in human cardiac patients (33).

To confirm our hypothesis that P_(a-ET)_CO_2_ and VDalv_frac_ are influenced by parameters determining lung perfusion even further, we tested the influence of CO and pulmonary arterial pressure, where especially high CO values increased P_(a-ET)_CO_2_ and VDalv_frac_. The influence of CO on the two CO_2_ indices was very likely due to its direct effect on venous admixture. It is known that increases in CO increase pulmonary pressure whereby pulmonary vessels perfusing collapsed lung areas are recruited. The blood flowing through these vessels increase venous admixture (34) also explaining why we saw an effect of pulmonary pressure on the VDalv_frac_.

Our results also confirm earlier findings in humans that both CO_2_ indices have a good correlation with Enghoff’s approach and derived variables (3). The fact that Enghoff’s approach represents pulmonary dead space as well as venous admixture is well accepted (10, 12, 16, 30, 31, 35). The fact, however, that venous admixture also affects P_(a-ET)_CO_2_ and VDalv_frac_ is not even well established in the human anesthesia literature (18, 30, 31) and has not made its way into clinical veterinary anesthesia. This is why the two CO_2_ indices are still frequently used in equine anesthesia to estimate pulmonary dead space (36). There are only a few papers specifically studying the two factors (6, 37–39). Most of the variables which have been shown to effect P_(a-ET)_CO_2_ and VDalv_frac_ in horses like duration of anesthesia, body weight, dorsal recumbency, and ventilation mode are known to influence venous admixture, CO, and/or pulmonary pressure. Our findings suggest that the idea of P_(a-ET)_CO_2_ or VDalv_frac_ being solely representative of dead space is erroneous or at least questionable in cases of high venous admixture typically observed in anesthetized horses.

The high correlation found in this study between the two CO_2_ indices with V/Q_Eng_ and V/Q_alv_Eng/VT_alv_ suggests that P_(a-ET)_CO_2_ or/and VDalv_frac_ might be good estimates of global V/Q mismatching in horses. This assumption is based on the fact that variables based on Enghoff’s approach have been shown to represent the sum of dead space and venous admixture (10, 16). This might provide equine anesthetist with a new clinical tool to evaluate the lung’s status; a high P_(a-ET)_CO_2_ or VDalv_frac_ with an adequate oxygenation (PaO_2_) might indicate a high physiological dead space, which points toward low CO and/or overdistension of lung tissue during controlled mechanical ventilation and recruitment maneuvers or even lung embolism (29, 40). On the other hand low PaO_2_ values in conjunction with a high P_(a-ET)_CO_2_ or/and VDalv_frac_ might point toward high $\dot{Q}s\text{​}/\text{​}\dot{Q}t$ probably in combination with high CO and/or pulmonary pressures as seen in our horses. However, further clinical validation in horses is required to support our assumptions.

We also evaluated the influence of other factors like VT, VO_2_, and SIII on the two CO_2_ indices as they might have caused changes in the arterial and end-tidal CO_2_ difference (14, 15, 19). None of the three parameters contributed any significant variance to either CO_2_ index. However, we did not see high variability in our data set for VO_2_ and SIII.

One limitation of our study is that VCap has not been directly validated in horses against the multiple inert gas elimination technique for the measurement of Bohr’s dead space in the same rigorous way as it has been done for pigs (35). However, as the principle of VCap is based on respiratory physiology, it can be applied to all mammals as long as the trachea and lung follow similar architectures. The special flow-partitioning device for large animals and mathematical correction for the splitting of the tidal volume into four equal parts has already been shown to provide valid VCap parameters in horses (22, 29, 40, 41).

Only six horses were included in this study as the number was set by the primary investigation (23). Hence, no power calculation was performed prior to commencing this study. However, we were able to answer our study question with the collected data sets specifically for healthy spontaneously breathing horses in dorsal recumbency. No inferences can be drawn on how pulmonary disease or mechanical ventilation would affect the two CO_2_ indices. Most importantly future studies in horses with higher alveolar Bohr’s dead space need to evaluate the influence of this V/Q mismatch “extreme” on the two studied CO_2_ indices.

The anesthetic protocol and inspired gas composition might have influenced the measurements and results might vary with different drug protocols and FiO_2_ level. Furthermore the hemoglobin analysis was performed with a photometric method not validated for whole horse blood, which might have induced an error in the absolute values of $\dot{Q}s\text{​}/\text{​}\dot{Q}t$ and VO_2_ reported here.

These limitations may have an impact on the predictor validity of the investigated parameters. Future studies should investigate the effect of the listed limiting factors influencing the measurements and their potential clinical influences on the two CO_2_ indices.

## Conclusion

Both CO_2_ indices, P_(a-ET)_CO_2_, or VDalv_frac_ represent global indices of the inefficiency of gas exchange and V/Q mismatching, and both are affected by venous admixture in healthy, anesthetised, spontaneously breathing horses. Under such conditions, the indices should not be used to estimate pulmonary dead space.

## Ethics Statement

This experimental study was carried out in accordance with the recommendations of the ARRIVE guidelines and had ethical approval by the local committee for animal experimentation of the Swiss government (TV-4985).

## Author Contributions

All authors contributed to conception and design of research. MM, UA, and RB performed experiments. AR, SB, GH, GT, and JS analyzed data and did statistical analysis. All authors interpreted results. MM, SB, AR, GT, and JS drafted manuscript. All authors edited, revised, and approved final version of the manuscript.

## Conflict of Interest Statement

The authors declare that the research was conducted in the absence of any commercial or financial relationships that could be construed as a potential conflict of interest.

## References

[1] Nyman G Funkquist B Kvart C Frostell C Tokics L Strandberg A Atelectasis causes gas exchange impairment in the anaesthetised horse. Equine Vet J (1990) 22(5):317–24. 10.1111/j.2042-3306.1990.tb04280.x 2226395

[2] Briganti A Portela DA Grasso S Sgorbini M Tayari H Bassini JR Accuracy of different oxygenation indices in estimating intrapulmonary shunting at increasing infusion rates of dobutamine in horses under general anaesthesia. Vet J (2015) 204(3):351–6. 10.1016/j.tvjl.2015.04.002 25920771

[3] Nunn JF Hill DW Respiratory dead space and arterial to end-tidal carbon dioxide tension difference in anesthetized man. J Appl Physiol (1960) 15:383–9. 10.1152/jappl.1960.15.3.383 14427915

[4] Severinghaus JW Stupfel MA Bradley AF Alveolar dead space and arterial to end-tidal carbon dioxide differences during hypothermia in dog and man. J Appl Physiol (1957) 10(3):349–55. 10.1152/jappl.1957.10.3.349 13438782

[5] Mitchell B Littlejohn A Influence of anaesthesia and posture on arterial oxygen and carbon dioxide tensions, alveaolr dead space and pulse rate in the horse. Vet Anaesth Analg (1972) 3(1):61–74.

[6] Moens Y Arterial-alveolar carbon dioxide tension difference and alveolar dead space in halothane anaesthetised horses. Equine Vet J (1989) 21(4):282–4. 10.1111/j.2042-3306.1989.tb02168.x 2504578

[7] McDonell WN Kerr C Physiology, pathophysiology, and anesthetic management of patients with respiratory disease. 5th ed In: Grimm KA Lamont LA Tranquilli WJ Greene SA Robertson SA, editors. Veterinary Anesthesia and Analgesia. Iowa, US: Wiley Blackwell (2015). p. 513–55.

[8] Bohr C Über die Lungenathmung. Centralblatt für Physiologie (1887) 1(14):236–68.

[9] Enghoff H Volumen inefficax. Bemerkungen zur Frage des schädlichen Raumes. Uppsala Lak Forhandl (1938) 44:191–218.

[10] Tusman G Sipmann FS Bohm SH. Rationale of dead space measurement by volumetric capnography. Anesth Analg (2012) 114(4):866–74. 10.1213/ANE.0b013e318247f6cc 22383673

[11] Wagner PD. Causes of a high physiological dead space in critically ill patients. Crit Care (2008) 12(3):148. 10.1186/cc6888 18492224 PMC2481441

[12] Suarez-Sipmann F Santos A Bohm SH Borges JB Hedenstierna G Tusman G. Corrections of Enghoff’s dead space formula for shunt effects still overestimate Bohr’s dead space. Respir Physiol Neurobiol (2013) 189(1):99–105. 10.1016/j.resp.2013.06.020 23827851

[13] Verscheure S Massion PB Verschuren F Damas P Magder S. Volumetric capnography: lessons from the past and current clinical applications. Crit Care (2016) 20(1):184. 10.1186/s13054-016-1377-3 27334879 PMC4918076

[14] Breen PH Mazumdar B Skinner SC. Comparison of end-tidal PCO_2_ and average alveolar expired PCO_2_ during positive end-expiratory pressure. Anesth Analg (1996) 82(2):368–73. 10.1097/00000539-199602000-00027 8561343

[15] Hardman JG Aitkenhead AR. Estimation of alveolar deadspace fraction using arterial and end-tidal CO_2_: a factor analysis using a physiological simulation. Anaesth Intensive Care (1999) 27(5):452–8. 10520383 10.1177/0310057X9902700503

[16] Suarez-Sipmann F Bohm SH Tusman G. Volumetric capnography: the time has come. Curr Opin Crit Care (2014) 20(3):333–9. 10.1097/MCC.0000000000000095 24785676

[17] Burrows FA. Physiologic dead space, venous admixture, and the arterial to end-tidal carbon dioxide difference in infants and children undergoing cardiac surgery. Anesthesiology (1989) 70(2):219–25. 10.1097/00000542-198902000-00007 2492409

[18] Strang CM Hachenberg T Freden F Hedenstierna G. Development of atelectasis and arterial to end-tidal PCO_2_-difference in a porcine model of pneumoperitoneum. Br J Anaesth (2009) 103(2):298–303. 10.1093/bja/aep102 19443420

[19] Tusman G Suarez-Sipmann F Bohm SH Pech T Reissmann H Meschino G Monitoring dead space during recruitment and PEEP titration in an experimental model. Intensive Care Med (2006) 32(11):1863–71. 10.1007/s00134-006-0371-7 17047925

[20] Douglas CG A method for determining the total respiratory exchange in man. J Physiol (1911) 42:17–8.

[21] Fletcher R. Deadspace during anaesthesia. Acta Anaesthesiol Scand Suppl (1990) 94:46–50. 10.1111/j.1399-6576.1990.tb03222.x 2291389

[22] Schramel JP Wimmer K Ambrisko TD Moens YP A novel flow partition device for spirometry during large animal anaesthesia. Vet Anaesth Analg (2014) 41(2):191–5. 10.1111/vaa.12099 24224723

[23] Mosing M MacFarlane P Bardell D Luthi L Cripps PJ Bettschart-Wolfensberger R. Continuous positive airway pressure (CPAP) decreases pulmonary shunt in anaesthetized horses. Vet Anaesth Analg (2016) 43(6):611–22. 10.1111/vaa.12357 26913706

[24] Kalchofner KS Picek S Ringer SK Jackson M Hassig M Bettschart-Wolfensberger R A study of cardiovascular function under controlled and spontaneous ventilation in isoflurane-medetomidine anaesthetized horses. Vet Anaesth Analg (2009) 36(5):426–35. 10.1111/j.1467-2995.2009.00477.x 19709046

[25] Kobler A Hartnack S Sacks M Bettschart-Wolfensberger R Evaluation of tidal volume measurements of the anaesthesia device Tafonius^®^ in vitro and in vivo. Pferdeheilkunde (2016) 32(5):449–56. 10.21836/PEM20160505

[26] Tusman G Scandurra A Bohm SH Suarez-Sipmann F Clara F. Model fitting of volumetric capnograms improves calculations of airway dead space and slope of phase III. J Clin Monit Comput (2009) 23(4):197–206. 10.1007/s10877-009-9182-z 19517259

[27] Lumb AB Nunn’s Applied Respiratory Physiology. 7th ed Oxford, England: Elsevier (2010).

[28] Blankman P Shono A Hermans BJ Wesselius T Hasan D Gommers D. Detection of optimal PEEP for equal distribution of tidal volume by volumetric capnography and electrical impedance tomography during decreasing levels of PEEP in post cardiac-surgery patients. Br J Anaesth (2016) 116(6):862–9. 10.1093/bja/aew116 27199318 PMC4872863

[29] Sacks M Mosing M Volumetric capnography to diagnose venous air embolism in an anaesthetised horse. Vet Anaesth Analg (2017) 44(1):189–90. 10.1111/vaa.12383 27174421

[30] Fletcher R Jonson B Cumming G Brew J. The concept of deadspace with special reference to the single breath test for carbon dioxide. Br J Anaesth (1981) 53(1):77–88. 10.1093/bja/53.1.77 6779846

[31] Drummond GB Fletcher R Deadspace: invasive or not? Br J Anaesth (2006) 96(1):4–7. 10.1093/bja/aei289 16357115

[32] Kerr CL McDonell WN Oxygen supplementation and ventilatory support. In: Muir WW Hubbell JAE, editors. Equine Anesthesia: Monitoring and Emergency Therapy. St. Louis, Missouri, US: Saunders Elsevier (2009). p. 332–52.

[33] Fletcher R. The relationship between the arterial to end-tidal PCO_2_ difference and hemoglobin saturation in patients with congenital heart disease. Anesthesiology (1991) 75(2):210–6. 10.1097/00000542-199108000-00007 1907112

[34] Benumof JL Wahrenbrock EA. Blunted hypoxic pulmonary vasoconstriction by increased lung vascular pressures. J Appl Physiol (1975) 38(5):846–50. 10.1152/jappl.1975.38.5.846 1126894

[35] Tusman G Sipmann FS Borges JB Hedenstierna G Bohm SH. Validation of Bohr dead space measured by volumetric capnography. Intensive Care Med (2011) 37(5):870–4. 10.1007/s00134-011-2164-x 21359609

[36] Robinson NE The respiratory system. In: Muir WW Hubbell JAE, editors. Equine Anesthesia: Monitoring and Emergency Therapy. St. Louis, Missouri: Saunders (2009). p. 11–36.

[37] Rainger JE Dart CM Perkins NR. Factors affecting the relationship between arterial and end-tidal carbon dioxide pressures in the anaesthetised horse. Aust Vet J (2010) 88(1–2):13–9. 10.1111/j.1751-0813.2009.00535.x 20148820

[38] Neto FJ Luna SP Massone F Thomassian A Vargas JL Junior JR The effect of changing the mode of ventilation on the arterial-to-end-tidal CO_2_ difference and physiological dead space in laterally and dorsally recumbent horses during halothane anesthesia. Vet Surg (2000) 29(2):200–5. 10.1111/j.1532-950X.2000.00200.x 10730713

[39] Meyer RE Short CE. Arterial to end-tidal CO_2_ tension and alveolar dead space in halothane- or isoflurane-anesthetized ponies. Am J Vet Res (1985) 46(3):597–9. 3922264

[40] Ambrisko TD Schramel J Hopster K Kastner S Moens Y. Assessment of distribution of ventilation and regional lung compliance by electrical impedance tomography in anaesthetized horses undergoing alveolar recruitment manoeuvres. Vet Anaesth Analg (2017) 44(2):264–72. 10.1016/j.vaa.2016.03.001 28237681

[41] Mosing M Auer U MacFarlane P Bardell D Schramel J Bohm SH Regional ventilation distribution and dead space in anaesthetised horses treated with and without continuous positive airway pressure: novel insights by electrical impedance tomography and volumetric capnography. Vet Anaesth Analg (2018) 45(1):31–40. 10.1016/j.vaa.2017.06.004 29222030

